# Thriving through transition: a narrative review of optimism, self-compassion, and academic stress in first-year college students

**DOI:** 10.3389/fpsyg.2026.1762426

**Published:** 2026-05-11

**Authors:** Nishanth Ghaty, Sanjana Vijayan, Ranjith Selvan, Sneha Srinivasan, Sandra Manoj, Maurvi Sonnegowda

**Affiliations:** School of Psychological Sciences, Christ University, Bangalore, India

**Keywords:** academic stress, first-year students, narrative review, optimism, self-compassion

## Abstract

**Introduction:**

Optimism and self-compassion are increasingly recognized as psychological resources relevant to academic stress regulation, yet their complementary roles among first-year university students remain theoretically underintegrated and empirically limited.

**Methods:**

This narrative review synthesizes empirical and theoretical research drawing on 44 peer-reviewed studies published between 2000 and 2024, employing structured search procedures to identify converging and divergent findings across four domains: direct associations, mediation and moderation processes, cultural and contextual influences, and intervention-related outcomes.

**Results:**

Across studies, both traits are generally associated with adaptive coping and lower perceived stress, although the strength and consistency of these relationships vary across contexts and study designs. Evidence regarding their interaction remains limited, with most findings derived from cross-sectional data. Cultural factors appear to shape how these traits are expressed and experienced, particularly in contexts where norms surrounding emotional expression and achievement differ.

**Discussion:**

Based on these patterns, the review proposes a dual-pathway conceptual framework distinguishing cognitive (optimism) and emotional (self-compassion) processes in academic stress regulation. This framework is intended as a heuristic model rather than an empirically validated mechanism. Methodological limitations, including reliance on cross-sectional designs and limited cultural diversity, are critically evaluated. The review highlights the need for longitudinal, culturally sensitive research to better understand these relationships and inform approaches to supporting student wellbeing during the first-year transition.

## Introduction

Academic stress is a prominent psychological concern in higher education, particularly among first-year undergraduate students. This stress arises from the convergence of intensive academic demands, unfamiliar institutional structures, and heightened personal and social expectations. Students often face sudden increases in workload, shifts toward self-directed learning, and identity redefinition in new academic and social environments. When these demands exceed perceived coping resources, psychological strain can result, as described by the cognitive-transactional model of stress (Lazarus and Folkman, 1984). Empirical research links academic stress to negative outcomes, including anxiety, depressive symptoms, burnout, and attrition (Beiter et al., 2014; Dyson and Renk, 2006; Eisenberg et al., 2007), which are considered related indicators of psychological strain within this review.

This vulnerability is especially pronounced during transitional phases such as the first year of university, where developmental instability intersects with academic responsibilities. The first-year transition represents a distinct developmental and contextual period characterized by shifts in autonomy, identity formation, and academic expectations, which may alter both the experience of stress and the effectiveness of coping resources. As such, findings derived from broader undergraduate populations may not fully capture the dynamics of this transitional phase. In collectivist cultural contexts, educational achievement is often seen as a moral duty linked to family reputation and social mobility, heightening internalized pressure and performance anxiety (Arimitsu, 2023; Deb et al., 2015; Pidgeon et al., 2014; Singh and Jha, 2013; Stoeber et al., 2019). Such environments can exacerbate maladaptive coping and compromise emotion regulation, highlighting the importance of considering cultural context when examining academic stress and its regulation.

Given these challenges, research has increasingly focused on internal psychological traits that promote resilience. Two constructs of particular interest are dispositional optimism—the tendency to expect positive outcomes (Seligman, 1998; Carver and Scheier, 2014)—and self-compassion—a mindful, kind, and accepting stance toward one's shortcomings (Neff, 2003, 2011). Both traits have been associated with improved emotion regulation, reduced stress, and enhanced motivation. However, findings are not uniformly positive, and the effectiveness of these traits appears to depend on contextual, cultural, and individual factors. Importantly, the common humanity component of self-compassion may be especially salient for first-year students navigating shared challenges and social comparison.

Despite a growing body of research, several limitations remain in the existing literature. First, optimism and self-compassion are often examined in isolation, with limited theoretical integration of their complementary roles in stress regulation, constraining the development of cohesive explanatory models. Second, relatively few studies explicitly situate these constructs within the first-year university transition, limiting understanding of how these processes operate within this distinct developmental context. Third, although cultural influences are increasingly acknowledged, they are rarely incorporated into integrative frameworks, limiting insight into how these traits function across diverse academic settings. Finally, variability in study designs, measurement approaches, and conceptual definitions has contributed to a fragmented understanding of the mechanisms linking these traits to academic stress.

To address these gaps, the present narrative review synthesizes empirical and theoretical research on optimism and self-compassion as psychological resources for regulating academic stress among first-year university students (rather than broader or mixed undergraduate populations). Rather than evaluating intervention efficacy, the review focuses on underlying psychological traits and mechanisms that shape students' responses to academic demands. Given the conceptual heterogeneity of constructs, variability in study designs, and the aim of developing an integrative theoretical framework, a narrative approach is more appropriate.

Building on this synthesis, the review proposes a dual-pathway framework in which optimism functions as a future-oriented cognitive resource influencing expectations and goal-directed behavior, while self-compassion operates as a present-focused emotional regulator that supports adaptive responses to failure and distress. This framework is theoretically grounded in existing empirical findings but has not yet been directly validated as an integrated model, and is therefore presented as a conceptual foundation to guide future research on student resilience, particularly in relation to developmental and cultural contexts.

## Theoretical background

### Optimism: a future-oriented coping resource with conditional efficacy

Optimism, broadly defined as the general expectation that positive outcomes will occur (Scheier and Carver, 1985; Seligman, 1998; Carver and Scheier, 2014), has been widely examined as a psychological trait associated with adaptive responses to stress. In academic contexts, particularly during the first-year university transition, optimism may function as a future-oriented cognitive resource, shaping appraisal processes, motivating goal-directed behavior, and potentially buffering students against academic stress. Optimistic students are more likely to interpret challenges as manageable, sustain engagement despite setbacks, and employ approach-oriented coping strategies such as planning and problem-solving (Chemers et al., 2001; Nes and Segerstrom, 2006; Reivich et al., 2012).

Two theoretical frameworks help contextualize optimism's function. Expectancy-Value Theory posits that individuals act when they perceive success as both possible and worthwhile; optimism may enhance expectancy, thereby promoting engagement. Conservation of Resources (COR) Theory conceptualizes optimism as a psychological resource that can support individuals in mobilizing coping strategies under stress, contributing to resilience (Carver et al., 2010; Hobfoll, 1989).

**Figure 1:**
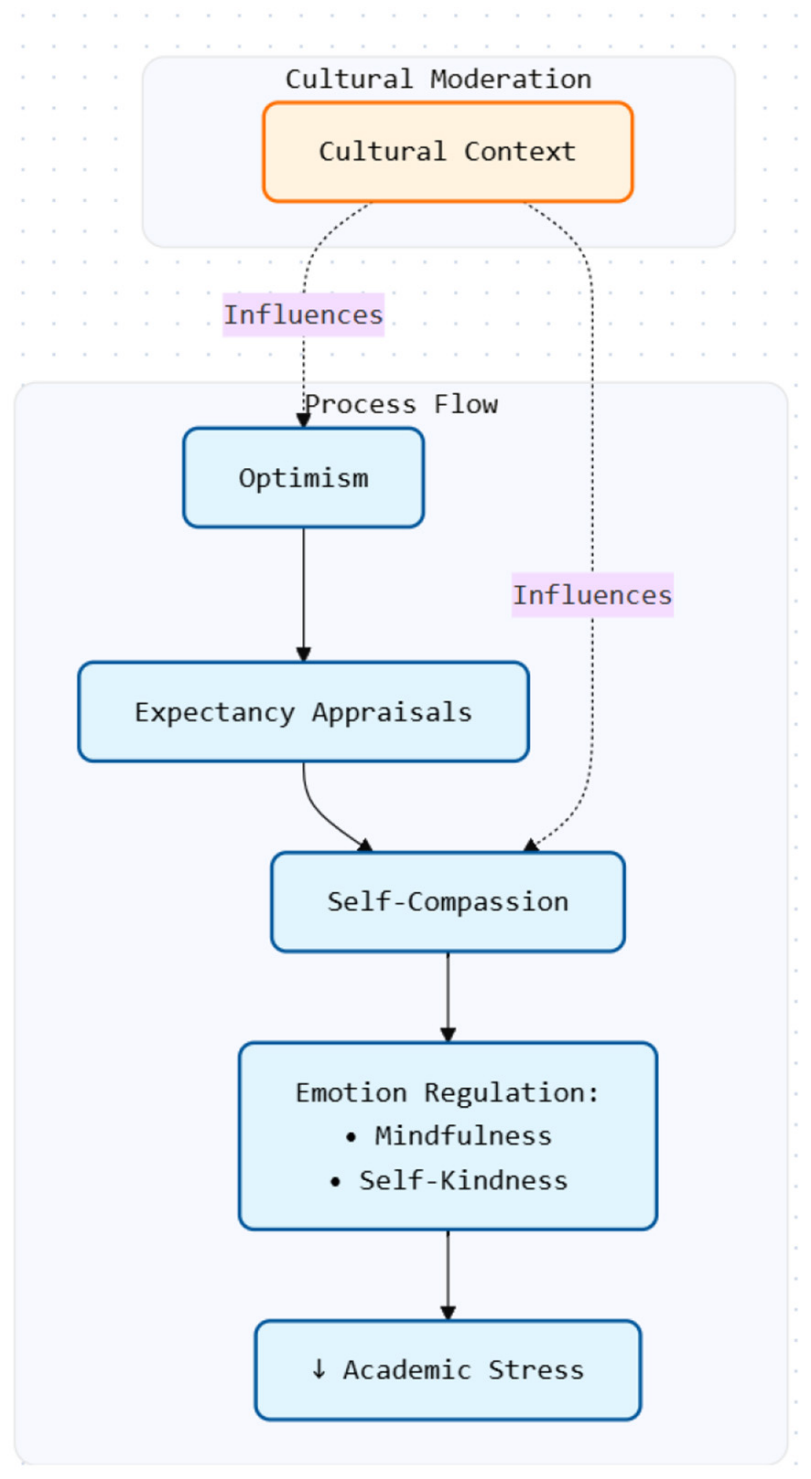
Conceptual model of optimism, self-compassion, and academic stress in first-year students.

Empirical research generally suggests that optimism is associated with lower academic stress, reduced emotional reactivity, improved adjustment, and higher academic persistence among first-year students (Aspinwall and Taylor, 1997; Chemers et al., 2001; Vizoso et al., 2019). However, these relationships are not uniformly observed. As seen in Figure 1, cultural and contextual factors may moderate its efficacy; for example, collectivist norms may constrain the expression of self-enhancing optimism or alter its relationship with coping and academic outcomes (Arimitsu, 2023; Singh and Jha, 2013).

While often adaptive, optimism may also have limitations. Excessive or unrealistic optimism can lead to overcommitment, inadequate preparation, or heightened stress when expectations are unmet (Shepperd et al., 2015). These findings suggest that the benefits of optimism are contingent on its calibration to situational demands.

### Self-compassion: a present-moment regulator of academic distress

Self-compassion refers to a mindful, kind, and accepting stance toward oneself in moments of difficulty or perceived failure (Neff, 2003; Neff and Germer, 2012). It comprises three interrelated components: self-kindness, common humanity, and mindfulness. Together, these facets support emotion regulation by enabling individuals to respond to stress with perspective and equanimity rather than self-criticism or avoidance. The common humanity component may be particularly relevant for first-year students, who are navigating shared academic challenges and social adjustment.

In higher education contexts, self-compassion has been associated with reduced academic stress, anxiety, and depressive symptoms, as well as more adaptive coping and cognitive reappraisal (MacBeth and Gumley, 2012; Raes, 2011; Zhang et al., 2016). It may enable students to maintain motivation and engagement without excessive rumination or perfectionism, distinguishing it from constructs such as self-esteem, which are more contingent on external validation (Neff and Vonk, 2008). However, findings are not entirely uniform. Some research suggests that increased awareness of distress associated with self-compassion may, in certain contexts, heighten the perception of stress, particularly in environments where emotional expression is constrained.

The effects of self-compassion are also shaped by contextual factors. Cultural norms may influence how self-kindness is interpreted or expressed; in some collectivist contexts, it may conflict with values emphasizing modesty, effort, or self-discipline (Arimitsu, 2023; Milyavskaya et al., 2018). Gendered expectations may further influence engagement with self-compassion practices, with some evidence suggesting both greater benefits and greater barriers among female students (Shirmohammadi et al., 2021; Smeets et al., 2014).

Intervention studies, including the Mindful Self-Compassion program (Neff and Germer, 2012), suggest that self-compassion can be cultivated and may contribute to improved wellbeing and reduced stress. In addition, self-compassion has been identified as a potential mediator in the relationship between other traits, such as optimism, and stress outcomes, indicating a possible role in translating cognitive expectations into emotional regulation processes.

### Dual pathways to resilience: the synergistic interaction of optimism and self-compassion

Although optimism and self-compassion are often examined independently, a growing body of research suggests that their combined influence may provide a useful framework for understanding how students respond to academic stress. Optimism may function as a future-oriented cognitive resource that supports goal-directed motivation and persistence, whereas self-compassion may operate as a present-focused emotional regulator that mitigates distress and preserves self-worth during setbacks (Carver and Scheier, 2014; Neff, 2003).

Together, these traits can be conceptualized as forming complementary processes that enable flexible responses to academic challenges. For example, following a disappointing academic outcome, optimism may support continued goal pursuit, while self-compassion may reduce self-criticism and facilitate emotional recovery. Empirical studies provide some support for this interaction; for instance, self-compassion has been shown to mediate the relationship between optimism and stress-related outcomes (Zhang et al., 2016; Zhao et al., 2022). However, existing studies have largely examined these relationships in isolation or through partial models, and evidence for their integrated functioning remains limited.

Some research suggests that the interaction between optimism and self-compassion may be synergistic rather than merely additive. Optimism in the absence of self-compassion may sustain motivation but leave individuals vulnerable to emotional distress, whereas self-compassion without optimism may support emotional stability but limit forward-directed engagement (Hope et al., 2014; Bui et al., 2021; Sujadi, 2022). These patterns point toward the potential value of considering both traits simultaneously.

**Figure 2:**
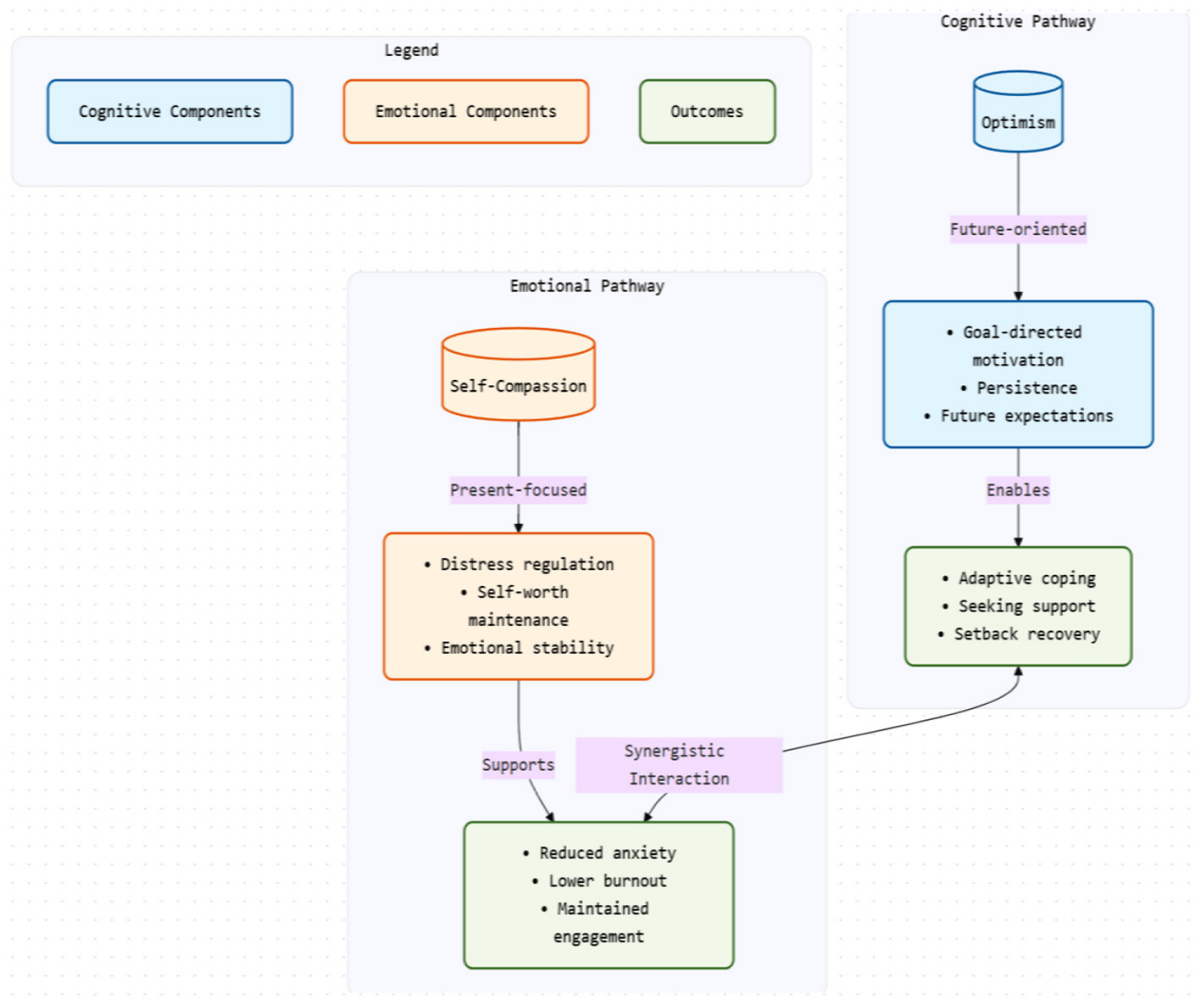
Proposed dual-pathway framework for academic stress regulation.

The dual-pathway framework proposed in this review builds on these insights by conceptualizing optimism as a cognitive pathway and self-compassion as an emotional pathway that jointly support adaptation during the first-year university transition. This framework is theoretically grounded in existing empirical findings but has not yet been directly validated as an integrated model. Figure 2 illustrates this proposed relationship. It is also important to note that this framework simplifies complex psychological processes into two primary pathways and may not fully capture the dynamic, reciprocal, and context-dependent nature of academic stress regulation.

Cultural and contextual factors are likely to shape this interaction. In individualistic settings, optimism and self-compassion may be more readily endorsed, whereas in collectivist contexts, norms surrounding achievement, endurance, and self-criticism may constrain or reshape how these traits are expressed and utilized (Arimitsu, 2023; Milyavskaya et al., 2018). Gendered expectations may further moderate these dynamics.

Taken together, these findings tentatively suggest that the effectiveness of optimism and self-compassion in regulating academic stress depends not only on their individual presence but also on their interaction and the broader cultural and developmental context in which they operate.

## Method

### Approach and rationale

The present review adopts a narrative approach to synthesize research on optimism, self-compassion, and academic stress among first-year undergraduate students. This approach was guided by established standards for narrative reviews, including principles outlined in the Scale for the Assessment of Narrative Review Articles (SANRA) (Baethge et al., 2019), with an emphasis on transparency, conceptual clarity, and structured synthesis.

Narrative reviews are particularly suited to topics characterized by theoretical and methodological diversity, as they allow for the integration of findings across study designs, conceptual frameworks, and cultural contexts (Sukhera, 2022). Unlike systematic reviews or meta-analyses, which prioritize methodological homogeneity and quantitative aggregation, narrative reviews enable the exploration of conceptual relationships, clarification of constructs, and identification of gaps in the literature. Given the conceptual heterogeneity of research on optimism, self-compassion, and academic stress—and the aim of developing an integrative theoretical framework—a narrative approach was considered more appropriate than a strictly quantitative synthesis.

While prior studies have examined optimism and self-compassion independently, relatively few have explored their combined roles in shaping academic stress regulation within the first-year university transition. Accordingly, this review aims to synthesize evidence across cognitive, emotional, and contextual dimensions to inform a theoretically grounded, but not yet empirically validated, dual-pathway framework.

### Search strategy

A structured literature search was conducted across PsycINFO, PubMed, Scopus, ERIC, and Web of Science. Google Scholar was used as a supplementary search tool for citation tracking. The search covered the period from 2000 to 2024. The lower bound reflects the introduction of self-compassion as a formal psychological construct (Neff, 2003), while the upper bound ensures inclusion of recent empirical and theoretical contributions.

Searches were conducted between October 2024 and March 2025 using Boolean combinations of keywords related to academic stress (e.g., “academic stress,” “university stress,” “student mental health”), population descriptors (e.g., “first-year students,” “freshman”), and psychological constructs (e.g., “dispositional optimism,” “self-compassion,” “emotion regulation,” “coping strategies”). Forward and backward citation tracking of seminal works (e.g., Carver and Scheier, 2014; Neff, 2003) was also performed to identify additional relevant studies. Google Scholar was included to enhance coverage and identify studies not indexed in primary databases, recognizing its broader scope but more limited reproducibility compared to curated academic databases.

Gray literature, including dissertations, conference proceedings, and non-peer-reviewed preprints, was excluded in order to prioritize peer-reviewed sources. This decision may introduce publication bias, which is acknowledged as a limitation.

### Eligibility criteria

Studies were considered eligible if they focused on first-year undergraduate students, examined outcomes related to academic stress, coping, adjustment, or resilience, and were published in English between 2000 and 2024.

Studies involving broader undergraduate samples were included only where participants were in early stages of university (typically aged 17–20) or where findings were explicitly linked to initial academic transition contexts (e.g., first-year adjustment or entry into higher education). Studies conducted in non-academic contexts were excluded unless findings were directly applicable to academic stress processes.

For the purposes of this review, “first-year students” were operationalized as individuals in their first 12 months of undergraduate enrolment. Studies including broader samples were retained only when findings were explicitly disaggregated or clearly attributable to this transition period. Interventions were considered only insofar as they provided insight into the modifiability or functional role of the focal traits, rather than as primary objects of evaluation.

Both empirical studies (quantitative, qualitative, and mixed-methods) and theoretical papers with substantive conceptual or analytical contributions were included in order to capture the breadth of relevant evidence. Theoretical papers were included only where they provided structured conceptual frameworks or integrative analyses grounded in existing literature, rather than purely opinion-based commentary. Studies were excluded if they focused exclusively on graduate or secondary school populations, consisted solely of opinion pieces, editorials, or commentaries without analytical or empirical grounding, or were not peer-reviewed.

This eligibility framework was designed to maintain a clear and coherent focus on the first-year university transition while allowing for limited inclusion of closely aligned populations where findings were directly relevant.

### Study selection process, data extraction, and management

Study screening was conducted by two reviewers using a structured process to enhance transparency and reduce selection bias. Discrepancies were resolved through discussion. Reference management and duplicate removal were facilitated using EndNote and Rayyan software. A total of 44 studies were included in the final synthesis. Consistent with narrative review methodology, these procedures were implemented to support transparency in study selection rather than to meet formal systematic review criteria.

Data extraction was conducted using a structured template capturing study characteristics, including author, year, country, sample size, study design, measures of optimism, self-compassion, and academic stress, and key findings.

Thematic domains were developed through iterative analysis of recurring patterns in the literature. These domains included direct associations, mediation and moderation processes, cultural and contextual influences, and intervention-related findings. As narrative synthesis prioritizes conceptual integration, studies were permitted to contribute to multiple thematic domains where relevant, rather than being restricted to mutually exclusive categories.

No formal quality appraisal or risk-of-bias assessment was conducted, consistent with the exploratory and theory-building aims of the review. However, study characteristics and methodological limitations (e.g., reliance on cross-sectional designs, self-report measures, and Western-centric samples) were considered during interpretation and are addressed in the discussion.

A summary of included studies is presented in Table 1.

**Table 1:** Summary of key studies on academic stress, optimism, and self-compassion.

Relationship type/Domain	Key representative studies	Key findings
1. Direct effects: optimism → ↓ academic stress	Carver and Scheier (2014); Chemers et al. (2001); Vizoso et al. (2019); Singh and Jha (2013); Peterson (2000); Nes and Segerstrom (2006)	Optimism is generally associated with lower perceived stress and more adaptive coping; however, findings are context-dependent. Some studies report increased anxiety under high-pressure conditions, particularly when optimism is unrealistic or poorly calibrated.
2. Direct effects: self-compassion (SC) → ↓ academic stress	Neff (2003); MacBeth and Gumley (2012); Hope et al. (2014); Terry et al. (2013); Sirois and Tosti (2012); Zhang et al. (2016); Sujadi (2022)	Self-compassion is frequently associated with lower stress, reduced self-criticism, and improved emotional regulation. However, effects may overlap with broader emotion regulation constructs, and may vary depending on cultural context and individual differences in emotional awareness.
3. Mediation: optimism → self-compassion → ↓ academic stress	Bui et al. (2021); Zhao et al. (2022); Hope et al. (2014); Neff and Germer (2012)	Evidence suggests that self-compassion may mediate associations between optimism and stress-related outcomes. However, most findings are based on cross-sectional designs, limiting conclusions about causal direction or temporal sequencing.
4. Mediation: coping → self-compassion → ↓ stress/anxiety	Smeets et al. (2014); Sujadi (2022); Bui et al. (2021); Zhang et al. (2016)	Adaptive coping strategies (e.g., mindfulness, proactive coping) appear more effective when associated with higher self-compassion. Directionality remains unclear, as self-compassion may function both as a mechanism and as an outcome of coping processes.
5. Moderation: self-compassion/optimism buffering negative traits (e.g., perfectionism, FoMO)	Stoeber et al. (2019); Milyavskaya et al. (2018); Circir and Tagay (2024); Gilbert et al. (2010)	Both traits have been shown to attenuate the impact of risk factors such as perfectionism and fear of missing out. However, moderating effects vary across studies and may depend on individual differences and contextual conditions.
6. Moderation: optimism or self-compassion as buffer under stress conditions	Kroshus et al. (2020); Naidoo and Oosthuizen (2023); Vizoso et al. (2019)	Higher levels of optimism and self-compassion are associated with fewer negative outcomes under elevated academic stress. Findings support a stress-buffering interpretation, though variability in measurement and design limits comparability.
7. Interactional or reciprocal influence: SC ↔ optimism	Circir and Tagay (2024); Hope et al. (2014); Sujadi (2022)	Preliminary evidence suggests a reciprocal relationship, where self-compassion may support more adaptive forms of optimism and vice versa. Longitudinal evidence remains limited, and findings should be considered exploratory.
8. Trait integration in transition support (Applied outcomes)	Conley et al. (2012); Ball et al. (2024); Pancer et al. (2000); Krieg (2013)	Both traits are associated with better adjustment, engagement, and wellbeing during the first-year transition. However, few studies directly examine developmental mechanisms or changes over time.
9. Cultural moderation of trait effects	Arimitsu (2023); Singh and Jha (2013); Zhao et al. (2022); González et al. (2015); Shirmohammadi et al. (2021)	Cultural context appears to shape both the expression and impact of optimism and self-compassion. Evidence from non-Western samples is limited, and findings suggest constructs may not operate equivalently across contexts.
10. Trait development through intervention	Smeets et al. (2014); Neff and Germer (2012); Reivich et al. (2012); Poots and Cassidy (2019)	Intervention studies suggest that optimism and self-compassion may be modifiable and associated with improved outcomes. However, studies vary in design, duration, and measurement, and mechanisms of change remain unclear.

### Synthesis

The synthesis integrates evidence across methodological approaches and conceptual domains to examine how optimism and self-compassion relate to academic stress in first-year university contexts. Rather than assuming uniform effects, both convergent and divergent findings are considered, with attention to contextual variability.

While the review engages with developmental considerations where relevant, the primary focus is on psychological mechanisms and contextual influences, reflecting the available evidence base. Cultural and gender-related moderators are incorporated where supported by the literature, but claims regarding generalizability are made cautiously.

The dual-pathway framework is used as an interpretive lens to organize findings, rather than as a pre-established or empirically validated model. This approach enables a structured synthesis while identifying areas where further empirical investigation—particularly longitudinal and culturally diverse research—is needed.

## Critical synthesis of existing research

The empirical literature examining optimism and self-compassion in relation to academic stress reflects a heterogeneous and methodologically uneven evidence base. While a substantial number of studies report associations consistent with a stress-buffering role for both traits, these findings vary in magnitude, context, and methodological robustness. Much of the existing research is cross-sectional, relies heavily on self-report measures, and is concentrated in Western university samples, which limits causal inference and generalizability. As such, the literature provides suggestive but not definitive evidence regarding how optimism and self-compassion function as protective psychological resources during the first-year university transition.

The association between optimism and academic stress is among the most extensively studied relationships. Theoretical models position optimism as a future-oriented expectancy that facilitates adaptive appraisal and persistence (Carver and Scheier, 2014). Empirical findings generally indicate that higher levels of optimism are associated with lower perceived stress, greater use of problem-focused coping, and improved academic adjustment (Chemers et al., 2001; Vizoso et al., 2019). Meta-analytic work (Nes and Segerstrom, 2006) supports a negative association between optimism and stress-related outcomes across contexts. However, the strength and consistency of these associations vary considerably. Many studies rely on correlational designs, making it difficult to determine whether optimism reduces stress, whether lower stress enhances optimism, or whether both are influenced by third variables such as socioeconomic context, prior academic achievement, or personality traits (e.g., neuroticism).

Moreover, emerging evidence complicates the assumption that optimism is uniformly beneficial. Singh and Jha (2013) reported a positive association between optimism and anxiety in high-pressure academic contexts, suggesting that optimism may, under certain conditions, reflect socially desirable responding or unrealistic expectations. Similarly, research on unrealistic optimism (Peterson, 2000; Shepperd et al., 2015) indicates that overly positive expectancies can reduce preparatory behaviors and increase vulnerability when outcomes do not align with expectations. These findings point to a more conditional interpretation in which optimism is adaptive primarily when it is calibrated to situational constraints and supported by effective coping strategies.

The literature on self-compassion presents a somewhat more consistent pattern of associations with reduced academic stress and improved emotional regulation. Self-compassion has been linked to lower levels of anxiety, depression, and self-criticism, as well as greater resilience and adaptive coping (Neff, 2003; MacBeth and Gumley, 2012; Zhang et al., 2016). These findings are theoretically coherent, given that self-compassion involves non-judgmental awareness and self-kindness in response to failure, which may reduce maladaptive rumination and emotional reactivity. However, similar methodological limitations apply. Much of the evidence is derived from cross-sectional designs, and the use of self-report scales for both predictor and outcome variables raises concerns about shared method variance.

In addition, the apparent consistency of self-compassion's benefits may partly reflect measurement and conceptual overlap with related constructs such as emotional regulation, mindfulness, and low self-criticism. This raises the possibility that self-compassion may function, in part, as a proxy for broader affective regulation capacities rather than a distinct mechanism. Some findings also suggest that increased awareness of distress associated with self-compassion practices may initially heighten perceived stress, particularly in contexts where emotional expression is discouraged or stigmatized. This indicates that the effects of self-compassion may be contingent on both individual readiness and sociocultural context.

A growing subset of studies has examined the relationship between optimism and self-compassion through mediation models, often positioning self-compassion as a mechanism through which optimism influences stress-related outcomes. Studies by Bui et al. (2021), Zhao et al. (2022), and Hope et al. (2014) report indirect effects consistent with this interpretation. However, these models are typically based on cross-sectional data, which limits the ability to establish temporal ordering or causal direction. It remains unclear whether optimism fosters self-compassion, whether self-compassion enhances optimistic outlooks, or whether both emerge from underlying regulatory capacities. As such, mediation findings should be interpreted as indicative of potential pathways rather than evidence of established mechanisms.

Research examining self-compassion as a mediator of coping processes further suggests that its effects may be embedded within broader regulatory systems. Studies indicate that mindfulness-based and proactive coping strategies are more strongly associated with reduced stress when accompanied by higher levels of self-compassion (Smeets et al., 2014; Zhang et al., 2016; Sujadi, 2022). While this supports the idea that self-compassion may enhance the effectiveness of coping strategies, it also raises questions about directionality. Self-compassion may be both an outcome of adaptive coping practices and a contributor to their effectiveness, making it difficult to disentangle cause and effect within existing study designs.

Moderation models provide additional insight but also reveal variability. Evidence suggests that both optimism and self-compassion may buffer the impact of risk factors such as perfectionism, fear of missing out, and high workload (Stoeber et al., 2019; Gilbert et al., 2010; Circir and Tagay, 2024; Naidoo and Oosthuizen, 2023). However, the strength of these moderating effects differs across studies, and the operationalization of both predictors and outcomes is not consistent. Differences in measurement, sample characteristics, and analytical approaches limit comparability and make it difficult to determine the conditions under which these buffering effects are most pronounced.

A smaller body of research has explored potential reciprocal or bidirectional relationships between optimism and self-compassion. Preliminary findings suggest that self-compassion may support the development of more realistic forms of optimism, while optimism may increase openness to self-compassionate perspectives (Circir and Tagay, 2024; Sujadi, 2022). Longitudinal evidence remains limited, however, and most studies do not directly test reciprocal models. As a result, claims regarding co-development or dynamic interaction between these traits remain speculative.

Evidence specific to the first-year university transition highlights the potential relevance of these traits for adjustment and engagement. Studies have linked optimism and self-compassion to improved wellbeing, persistence, and academic adaptation during this period (Conley et al., 2012; Ball et al., 2024). At the same time, research on transition-related stress emphasizes the role of expectation–reality mismatches in shaping adjustment difficulties (Pancer et al., 2000; Krieg, 2013). While these findings suggest that optimism and self-compassion may be relevant to transitional processes, relatively few studies explicitly examine developmental mechanisms or track changes in these traits over time. Consequently, conclusions about their developmental role during the first-year transition remain limited.

Cultural factors introduce further complexity. Much of the existing literature is based on Western samples, where constructs such as optimism and self-compassion are culturally reinforced. In collectivist contexts, these traits may be interpreted differently or may interact with norms emphasizing effort, modesty, or social obligation. For example, Arimitsu (2023) reported lower levels of self-compassion among Japanese students, while Singh and Jha (2013) found that optimism was associated with increased anxiety in an Indian academic context. These findings suggest that the meaning and function of these constructs may not be culturally invariant. However, the limited number of non-Western studies constrains the ability to draw robust cross-cultural conclusions, and existing findings should be interpreted cautiously.

Intervention-based studies provide preliminary evidence that both optimism and self-compassion can be enhanced through structured programs (e.g., Smeets et al., 2014; Neff and Germer, 2012; Reivich et al., 2012), with reported improvements in wellbeing and academic functioning. However, these studies vary widely in design, duration, and outcome measures, and often lack long-term follow-up. In addition, intervention effects may reflect broader factors such as increased attention, social support, or engagement with reflective practices, rather than changes in the targeted traits alone. As such, while these findings suggest modifiability, they do not provide conclusive evidence regarding the mechanisms through which such changes occur.

Taken together, the literature indicates that optimism and self-compassion are associated with adaptive responses to academic stress, but these relationships are neither uniform nor fully understood. Evidence for their interaction is suggestive but remains methodologically limited, and the extent to which these traits operate synergistically has not been definitively established. Variability in study design, measurement, and cultural context further complicates interpretation. Future research would benefit from longitudinal designs, multi-method approaches, and greater cultural diversity to more precisely delineate the conditions under which these traits support resilience during the first-year university transition.

## Results

The literature included in this review (*N* = 44) reflects a heterogeneous set of findings regarding the relationships between optimism, self-compassion, and academic stress among first-year university students. While several recurring patterns can be identified, the strength and consistency of these relationships vary across studies, contexts, and methodological approaches. For clarity, findings are organized into four domains: direct effects, mediation and moderation, interaction and reciprocal effects, and transition, contextual, and intervention-related outcomes. Table 1 summarizes the included studies and their primary relationships.

As illustrated in Figure 2, these patterns can be organized into a conceptual dual-pathway framework linking cognitive (optimism) and emotional (self-compassion) processes to academic stress regulation. This figure represents a heuristic integration of observed associations across studies rather than an empirically validated or causal model. The directional relationships depicted are intended to aid conceptual organization and should not be interpreted as definitive causal pathways.

### Direct effects

A number of studies report associations between optimism and reduced academic stress, as well as improved coping and persistence (Carver and Scheier, 2014; Chemers et al., 2001; Vizoso et al., 2019). However, these relationships are not uniformly observed. Some findings suggest that in high-pressure academic environments, optimism may be associated with increased anxiety, particularly when expectations are unrealistic or misaligned with situational demands (Singh and Jha, 2013).

Similarly, self-compassion has been associated with lower levels of stress, reduced self-criticism, and greater emotional resilience in response to academic challenges (Neff, 2003; MacBeth and Gumley, 2012; Zhang et al., 2016). While these associations appear relatively consistent across studies, they are largely derived from cross-sectional and self-report data, which limits causal interpretation and raises the possibility of shared method variance.

### Mediation and moderation

Several studies suggest that self-compassion may function as a mediating variable linking optimism or coping behaviors to academic stress outcomes (Bui et al., 2021; Smeets et al., 2014; Zhao et al., 2022). These findings indicate that reductions in stress may be more likely when cognitive expectations are accompanied by effective emotional regulation processes. However, most mediation models are based on cross-sectional designs, and therefore cannot establish temporal or causal pathways.

Moderation effects have also been reported, with both optimism and self-compassion associated with reduced impact of psychological risk factors such as perfectionism and fear of compassion (Gilbert et al., 2010; Stoeber et al., 2019). The strength and consistency of these effects vary across studies, and differences in measurement and sample characteristics limit comparability.

### Interaction and reciprocal effects

Some studies propose that optimism and self-compassion may be interrelated, with each trait potentially reinforcing the other (Circir and Tagay, 2024; Hope et al., 2014; Sujadi, 2022). For example, optimism may be associated with greater openness to self-compassionate responses, while self-compassion may support more adaptive or realistic forms of optimism. This conceptual relationship is reflected in Figure 2 as a potential interaction between cognitive and emotional pathways. However, empirical evidence for such interaction remains limited, and most studies do not directly test bidirectional or longitudinal models. These findings should therefore be interpreted as preliminary.

### Transition, contextual, and intervention-related outcomes

Evidence suggests that both optimism and self-compassion are associated with aspects of adjustment during the first-year university transition, including wellbeing, engagement, and perceived academic competence (Conley et al., 2012; Ball et al., 2024). However, relatively few studies directly examine developmental processes or changes in these traits over time, limiting conclusions about their role in developmental adaptation.

Contextual and cultural factors also appear to influence these relationships. In collectivist or high-pressure academic environments, the expression and effects of optimism and self-compassion may differ, with some studies reporting attenuated or counterintuitive associations (Arimitsu, 2023; Singh and Jha, 2013; Zhao et al., 2022). However, the evidence base remains limited in its cultural diversity. These interpretations are exploratory and not grounded in a formal cross-cultural framework, reflecting limitations in the available evidence.

Intervention-based studies suggest that optimism and self-compassion may be modifiable through structured programs (Neff and Germer, 2012; Smeets et al., 2014; Poots and Cassidy, 2019). While these studies report improvements in wellbeing and academic functioning, variability in intervention design, duration, and outcome measurement, as well as limited long-term follow-up, constrain the strength of these conclusions. These findings are included to contextualize the applied relevance of these traits, rather than to evaluate intervention efficacy.

## Discussion

This review examined how dispositional optimism and self-compassion are associated with academic stress among first-year university students, with particular attention to their potential independent and joint roles as protective psychological resources. The synthesis of existing literature reveals a conceptually rich but empirically uneven evidence base, characterized by relatively consistent associations for each trait individually, but more limited and methodologically constrained evidence regarding their integration. The dual-pathway framework proposed in this review organizes these patterns by distinguishing between cognitive (optimism) and emotional (self-compassion) processes, while recognizing that their interaction is likely context-dependent and not yet empirically established as a unified mechanism.

At a conceptual level, optimism and self-compassion appear to occupy complementary psychological domains. Optimism is typically framed as a future-oriented expectancy shaping goal pursuit and persistence, whereas self-compassion is more closely linked to present-focused emotional regulation and responses to failure. This distinction is broadly reflected in the literature, with optimism associated with persistence, academic self-efficacy, and proactive coping (Carver and Scheier, 2014; Chemers et al., 2001), and self-compassion associated with reduced stress, lower self-criticism, and improved emotional regulation (Neff, 2003; MacBeth and Gumley, 2012; Sirois and Tosti, 2012). However, these associations should be interpreted cautiously, as much of the supporting evidence is cross-sectional and may reflect shared variance with broader constructs such as affect regulation and personality.

Evidence regarding the integration of these traits remains tentative. Mediation studies (e.g., Zhao et al., 2022; Bui et al., 2021) suggest that self-compassion may be involved in pathways linking optimism to stress-related outcomes, but such findings are largely derived from cross-sectional models and do not establish causal direction. It remains unclear whether optimism facilitates self-compassion, whether self-compassion enhances adaptive forms of optimism, or whether both reflect underlying regulatory capacities. Moreover, the direct association between optimism and reduced academic stress appears inconsistent, particularly in high-pressure contexts where unrealistic expectations may contribute to increased distress (Shepperd et al., 2015; Singh and Jha, 2013). These findings suggest that optimism's adaptive value may depend on contextual calibration rather than functioning as a uniformly protective trait.

Self-compassion demonstrates more consistent associations with reduced stress and maladaptive coping, but these findings are not without limitations. The apparent robustness of self-compassion effects may partly reflect conceptual and measurement overlap with constructs such as mindfulness and emotion regulation. Additionally, increased awareness of distress associated with self-compassion may, in some contexts, heighten perceived stress, particularly in environments where emotional expression is constrained (Zhang et al., 2016). These nuances indicate that the effectiveness of self-compassion is likely contingent on both individual and contextual factors.

From a developmental perspective, the first-year university transition provides a relevant but underexamined context for these processes. This period is characterized by increased academic demands, social reorientation, and identity-related challenges, which may amplify both stress and the potential relevance of adaptive psychological traits. While several studies link optimism and self-compassion to adjustment outcomes during this period, relatively few explicitly examine developmental trajectories or changes in these traits over time. As a result, conclusions regarding their role in developmental adaptation remain limited.

Cultural variability further complicates interpretation. Much of the existing literature is based on Western, individualistic samples, where both optimism and self-compassion are culturally reinforced. Evidence from collectivist contexts suggests that these constructs may function differently, and in some cases may be associated with increased stress due to cultural norms emphasizing self-criticism, endurance, or social obligation (Arimitsu, 2023; Singh and Jha, 2013). However, the limited representation of non-Western samples constrains the extent to which culturally grounded conclusions can be drawn.

Taken together, these findings suggest that while optimism and self-compassion are associated with adaptive responses to academic stress, their effects are neither uniform nor fully understood. The dual-pathway framework proposed in this review provides a useful conceptual lens for organizing these relationships, but should be understood as a heuristic model rather than an empirically validated system. Further research is needed to clarify the conditions under which these traits operate independently, interact, or co-develop over time.

### Gaps in the literature and future directions

The present review highlights several key gaps in the literature. First, the predominance of cross-sectional designs limits causal inference and obscures the temporal dynamics of optimism, self-compassion, and academic stress. While mediation models suggest potential pathways, they do not establish whether changes in one construct lead to changes in another. Longitudinal, experience sampling, and growth-curve methodologies are needed to better capture these dynamics and test developmental hypotheses.

Second, mechanistic pathways remain insufficiently specified. Although mediation and moderation models provide initial insights, few studies examine more complex or reciprocal relationships between optimism and self-compassion. Future research employing structural equation modeling, longitudinal mediation, or latent growth approaches could help clarify whether these traits operate as independent resources, mutually reinforcing processes, or components of broader regulatory systems.

Third, cultural and contextual limitations restrict generalizability. The overrepresentation of Western samples and psychology student populations limits the applicability of findings to more diverse educational and cultural contexts. Future work should prioritize culturally sensitive measurement, cross-cultural comparisons, and contextually grounded research designs.

Fourth, developmental specificity remains underexplored. Although the first-year transition is frequently invoked as a high-risk period, few studies directly examine how optimism and self-compassion evolve during this phase or interact with developmental processes such as identity formation and social comparison. Research focusing explicitly on first-year cohorts, with repeated measurement over time, would strengthen the developmental relevance of this literature.

Finally, while intervention studies suggest that both traits may be modifiable, the evidence base remains limited and heterogeneous. Few studies have examined interventions targeting both optimism and self-compassion simultaneously, and outcomes beyond psychological wellbeing—such as academic persistence and long-term adjustment—are rarely assessed. Future research should investigate multi-component interventions and evaluate their effects across diverse student populations and contexts.

## Conclusion

Academic stress remains a significant challenge in higher education, particularly during the first-year transition. This review synthesized existing research on optimism and self-compassion, highlighting their potential roles as psychological resources associated with stress regulation and resilience. While each trait is linked to adaptive outcomes, the nature of their interaction remains incompletely understood.

The dual-pathway framework proposed in this review offers a conceptual approach to organizing these relationships by distinguishing between cognitive and emotional processes. However, this framework should be interpreted cautiously, as current evidence does not establish a unified or causal model. Limitations in study design, cultural representation, and developmental focus constrain the strength of existing conclusions.

Future research adopting longitudinal, culturally sensitive, and methodologically rigorous approaches will be critical in advancing understanding of how these traits contribute to academic resilience. Such work has the potential to inform more targeted and contextually grounded approaches to supporting student wellbeing and adjustment in higher education.

## Data Availability

The original contributions presented in the study are included in the article. Further inquiries can be directed to the corresponding author.
